# An Overview of Systematic Reviews and Meta-Analyses on the Effect of Medication Interventions Targeting Polypharmacy for Frail Older Adults

**DOI:** 10.3390/jcm12041379

**Published:** 2023-02-09

**Authors:** Aparna Verma, Sanjib Saha, Johan Jarl, Ellen Conlon, Bernadette McGuinness, Dominic Trépel

**Affiliations:** 1 Health Economics Unit, Department of Clinical Science (Malmö), Lund University, SE-22381 Lund, Sweden; 2 Trinity College Institute for Neuroscience, Trinity College Dublin, D02 PN40 Dublin, Ireland; 3 Centre for Public Health, Queen’s University Belfast, Belfast BT12 6BA, UK; 4 School of Medicine, Trinity College Dublin, D02 R590 Dublin, Ireland

**Keywords:** frailty, polypharmacy, medication interventions

## Abstract

Frailty refers to the lack of resilience and a reduction in a person’s ability to recover following a health problem, and it is increasingly becoming a challenging aspect of ageing populations. Many older adults are exposed to polypharmacy; i.e., they continue to be on medications without timely re-evaluation. Medication reviews have proven successful in managing polypharmacy in the general population, but there is uncertainty regarding their effect among frail older adults. This overview of published systematic reviews assesses the impact of medication reviews on polypharmacy in frail older adults. Embase was searched from its inception to January 2021 and 28 systematic reviews were identified, out of which 10 were included in the overview. Medication reviews were the most common intervention in 8 out of 10 systematic reviews. The frailty score was reported as an outcome in one systematic review that found no evidence for fundamental pharmacological effects on frailty. Six systematic reviews reported a statistically significant reduction in the number of inappropriately prescribed medications. Four systematic reviews reported on hospital admissions, with two of them reporting a decrease in hospitalisations. The quality assessment was moderate in six and critically low in four of the systematic reviews. We conclude that medication reviews help in reducing the use of inappropriate medications in frail older adults, but that there is insufficient evidence in terms of frailty score and hospital admissions.

## 1. Introduction

Across the world, the population’s ageing is accelerating at a fast rate, from approximately a 461 million people aged over 65 years in 2004 to an estimated 2 billion people by the year 2050 [1]. The ageing of the population is a leading demographic phenomenon of the 21st century, which signifies the massive achievements of medical science, public health, and economic growth. It is a story of triumph as people today are living longer and continue to live relatively healthier lives compared to past centuries. However, the continual growth of the older population also comes with its own challenges. The ageing of the population affects the capability of states to deliver necessary resources, in terms of fiscal strains not only on pension and insurance systems (increased health care costs) but also on the social support systems [2]. As people are living longer, there is debate pertaining to the quality of life in the later years of a person’s life. Questions arise regarding their physical and mental health and whether older adults will enjoy sustained good health or if the later years will be marked by chronic maladies.

### 1.1. Frailty

The clinical condition of frailty is increasingly becoming the most challenging aspect of the ageing of the population. Frailty is defined as ‘a state of increased vulnerability to poor resolution of homeostasis following a stress’ [1], and it develops due to age-related degeneration across various physiological systems of the human body. The lack of resilience results in susceptibility to sudden changes in health status caused by relatively insignificant stressors. According to an estimate, nearly one-fourth to half of all individuals over the age of 85 years are frail and have a considerably greater probability of falls, disability, long-term care, and death [1]. A seemingly small change, for example, a new drug, a trivial infection, or a minor surgery, has the power to bring about intense and incommensurate changes in the health condition, from being independent to dependent, articulate to hallucinatory, or mobile to immobile. Though frailty is a dynamic process, there is a greater possibility of transitioning to more severe states than recovering [1].

### 1.2. Polypharmacy

Polypharmacy has been classified as a geriatric syndrome commonly present in older adults. It is defined as the use of multiple medications (often subjectively described as either the use of more than or equal to 5 or 10 medications for more than 90 days) [3]. The association of polypharmacy with adverse health outcomes, such as falls, adverse drug reactions, functional impairment, longer duration of stay in the hospitals, frequent readmissions, and mortality, makes it a matter of serious concern [4]. The adverse outcomes could possibly occur due to various factors that have a positive association with polypharmacy such as interactions between two drugs, interactions between drugs and diseases, or potentially inappropriate prescriptions. Hence, polypharmacy is increasingly being considered an important challenge in clinical practice [5]. It is also important to note that the incidence and prevalence of polypharmacy is high among older adults [3]. Many older adults continue to be on treatment without timely re-evaluation of the benefit/risk trade-off. Consequently, polypharmacy also impacts the sustainability of the public health care system both directly (e.g., compensation of costs for the use of unnecessary medications) and indirectly (e.g., through increased need for care following adverse effects) [6].

### 1.3. Frailty and Polypharmacy

Frailty and polypharmacy are both common and have been extensively studied in older adults, and in the last few years, some studies have tried to gauge and determine the relationship between them. The linkages via which frailty and polypharmacy can interact include biological alterations, numerous pathologies and chronic ailments, life expectancy, and cognitive status [5]. Notwithstanding the fact that there exists a noticeable association between the two, it is challenging to ascertain causality and establish which happens first, frailty or polypharmacy [6].

Numerous factors such as weight loss, poor nutrition, and balance disorders that can be measured as characteristics of frailty have been found to be associated with the number of medicines consumed [5]. The current state of knowledge does not make it evident which of these factors is involved in the development of frailty associated with polypharmacy. Some studies also indicate that frailty and polypharmacy may act as modulators for their harmful influence on health, so their interaction could also help establish the frequency of adverse health events [7,8]. This raises the question of whether frailty can be reduced by reducing the incidence and prevalence of polypharmacy.

Medication review is a type of medication intervention, defined as a systematic examination of a patient’s medicines with the purpose of augmenting the impact of medicines, reducing the number of medication-related complications, and decreasing waste [9], and it is a common practice to address polypharmacy. This is mostly performed by pharmacists independently or in a team with other health care professionals. Medication interventions have been shown to be a successful way of managing polypharmacy and decreasing inappropriate prescriptions in the general population [10]. There are many systematic literature reviews highlighting the effectiveness of medication interventions targeting polypharmacy in older adults, but they have provided mixed results. The intervention also varies, as it may be pharmacist-led, computer-based, or provided by introducing screening tools [11]. It is currently unknown if the intervention also is effective for frail older adults in terms of reducing frailty, health care utilisation, and inappropriate medication.

### 1.4. Aim

The aim of this overview of systematic reviews is to assess and summarise how medication interventions that target polypharmacy affect frail older adults in terms of frailty, inappropriate medications, and health care utilisation.

## 2. Materials and Methods

This overview is a part of a broader overview titled ‘An overview of systematic reviews for older people who become frail.’ The protocol of the overview is registered on the International Prospective Register of Systematic Reviews PROSPERO with Registration number CRD42021225390 [12]. The overview was conducted in two stages.

### 2.1. Stage 1

The search was conducted in EMBASE, and the search string was developed together with an expert librarian. The search string is presented in the supplementary material together with the PICO criteria. One of the criteria for ‘Population’ in PICO is that studies need to be about the Frailty syndrome described by the British Geriatric Society (BGS) [13]. No lower limit was placed on the publication date. The upper limit on the publication date was January 2021. Only SRs and meta-analyses in English were considered. Covidence software was used for screening and study selection. Screening was carried out by all members of the research team (6 members), blinded to each other’s decisions, with two matching votes required for a record to be included or excluded. Conflicts were resolved by group discussion.

### 2.2. Stage 2

**Search strategy:** For the second stage of the overview, studies that were tagged by at least one member of the research team during Stage 1 were reported as interim results (see Table S1 in the Supplementary Materials), and individual members of the research team then selected studies relevant to their specific research question. 

The research question presented here related to studies examining ‘*susceptibility to side effects of medication*’ and was primarily considering ‘*randomised controlled trials’* (as the study type); however, to ensure potentially relevant reviews were not missed, ‘other systematic reviews’ were also appraised. The reviews that were potentially relevant to our research question were then exported back into Endnote, and subsequently, Covidence and full-text files were obtained and screened for suitability against the following criteria. 

**Inclusion and Exclusion criteria:** The inclusion and exclusion criteria for this overview were formulated keeping in line with the research aim and following the population, intervention, comparison, outcome, study design and setting (PICOSs) reporting structure. Specific inclusion criteria are as follows:

- **Population**: Reviews must include studies with a focus on frailty or one of the frailty syndromes (Table S1) in adults over the age of 60 years.
- **Intervention**: Reviews must evaluate strategies targeting polypharmacy
- **Comparator**: Reviews must include studies comparing doing nothing, usual care, or the best alternative.
- **Outcomes**: Reviews must include either any outcomes considered clinically relevant or any intermediate outcomes (e.g., laboratory results) with well-established connections to frailty, health care utilisation, or inappropriate medication; studies specifically measuring change in frailty (on a recognised scale) were documented for reporting the proportion and potential subgroup analysis;
- **Study design**: Only systematic reviews of Randomised Controlled Trials were included.
- **Settings**: All healthcare settings were included (i.e., hospital and community-based interventions).

Studies were excluded if they were not written in English, or if they were commentaries, or letters to the editor.

**Figure 1:**
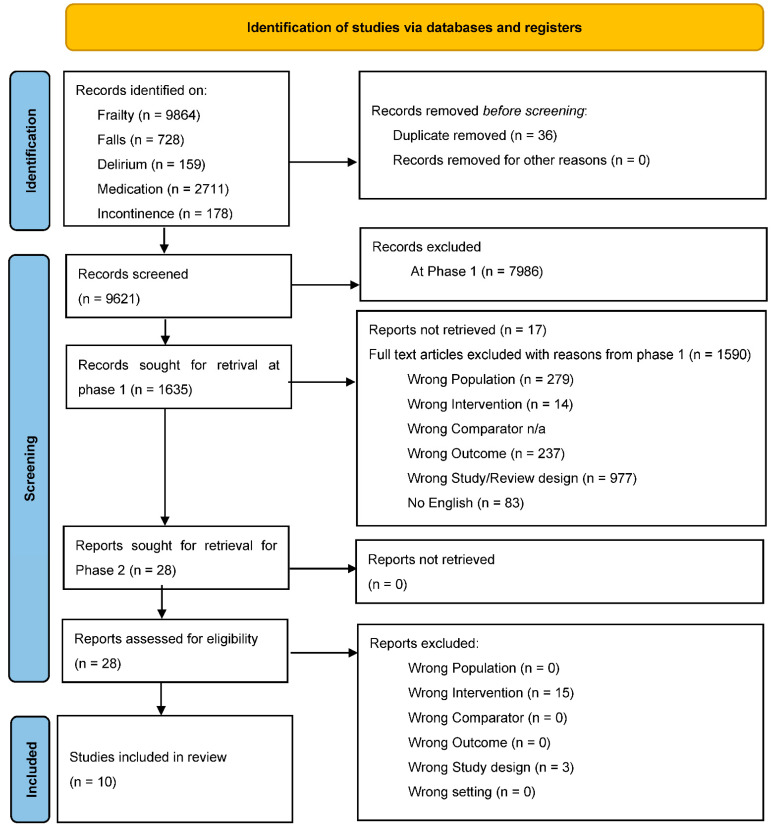
Prisma flow diagram.

**Screening**: The papers were assessed based on title/abstracts and full text, according to the inclusion and exclusion criteria specific to the aim of this overview. The Prisma flow diagram for Stage 2 (Figure 1) details the process in which the studies were identified, screened, and included in this review. 

**Data extraction**: Data extraction of the systematic reviews was carried out using an individualised data extraction form on Microsoft Excel. The data extraction sheet organised general information such as the year of publication, the aim of the study, countries in which the primary studies were conducted, names of databases searched, number of included studies that were randomised controlled trials (RCTs), number and age of participants, methodological considerations, and overall conclusions from the systematic reviews and data from the meta-analyses. 

### 2.3. Methodological Quality of Systematic Reviews

A quality assessment was completed for each systematic review using the AMSTAR 2 checklist [14]. This tool comprises a 16-item checklist covering significant domains to determine the quality of the systematic review. There are seven critical domains in the AMSTAR 2 checklist that are considered more important than others in affecting the validity of a review. The AMSTAR 2 tool categorises systematic reviews into ‘High quality’, ‘Moderate quality’, ‘Low quality’, and ‘Critically low quality’.

## 3. Results

### 3.1. Stage 1

The final search for Stage 1 was conducted in January 2021, which gave 9657 articles in total. Once uploaded to Covidence, 36 duplicates were removed, and the screening process was started on the remaining 9621 articles. A total of 1635 papers proceeded to the full-text screening and were assessed for eligibility, and any reason for exclusion was noted (Figure 1).

### 3.2. Search Results for Stage 2

A total of 28 studies were selected from Stage 1 for the current overview and screened and assessed for eligibility in Stage 2. It was noted that 15 had the wrong intervention (1 on medical adherence and 14 on individual medications) and 3 had the wrong study design (1 study protocol, 1 overview of reviews, and 1 review not of RCTs), leaving 10 systematic reviews that fulfilled the inclusion criteria. However, further discussion is required about whether the reviews used objective outcomes of frailty (see Discussion). As mentioned above, Stage 1 results with respect to types of included studies are available in (Table S1 in the Supplementary Material) and, in line with reporting standards, are summarised above in terms of what was included in Stage 2. 

### 3.3. Summary of Findings of Included Systematic Reviews

We summarise the key aspects of the included systematic reviews in terms of five characteristics. 

**Country population:** The countries of individual studies in the reviews were as follows: 31 studies from the USA; 17 studies each from the UK and Australia; 6 from Canada; 4 from Sweden; 3 each from Spain and Denmark; 2 each from Israel, Switzerland, Ireland, Norway, New Zealand, and Germany; and 1 each from India, Portugal, Jordan, Netherlands, Finland, and Singapore. 

**Intervention type:** The interventions used were medication reviews in 8 out of 10 studies, while the other two had electronic/computer tools for deprescribing as the intervention.

**Outcomes and meta-analysis:** Most of the studies included more than one outcome measure. The most common outcome measure was inappropriate medication (n = 6), followed by health care utilisation (n = 4) and frailty score (n = 1). Five studies also performed a meta-analysis on unplanned hospitalisation (n = 4) and prescription of inappropriate medication (n = 1). Of the outcome measures found in the included systematic reviews, no review included a meta-analysis on frailty status, and only 1 review had frailty as the primary outcome.

**Quality**: Risk of bias assessment of the studies with meta-analyses was conducted using the Cochrane Risk of Bias tool in three reviews and the EPOC risk of bias tool in one, and one review did not perform any risk of bias assessment. Of the five studies that did not conduct meta-analyses, two reviews conducted risk of bias assessment, and both found that the included trials were of low methodological quality. 

**Year:** Nine out of the ten included reviews were published in the last decade, and seven were published in the last two years. 

### 3.4. Summary of Included Systematic Reviews

The following summary of the included systematic reviews is presented in reverse chronological order.

**Tasai et al. [15], published in 2021**, aimed to evaluate the impact of medication reviews delivered by community pharmacists to older patients with polypharmacy in 4633 participants with a mean age of 74 years. The review included four randomised controlled trials and the following outcomes: hospitalization, emergency department (ED) visits, quality of life, and medication adherence. It concluded that clinical medication reviews for older people with polypharmacy reduced the risk of hospitalisation and ED visits. Three studies reported quality of life as an outcome with inconsistent results. Two studies used the SF-36 questionnaire for the measurement of quality of life and observed no substantial differences between the intervention and control groups, while the third study used the EuroQol-5D-3L questionnaire and reported an improvement in quality of life in the intervention group. Only one RCT reported on medication adherence, which was reported to be higher in intervention group.

**Abbot et al. [16], published in 2020,** assessed whether pharmacist home visits for medication reviews were successful in improving the health of individuals at risk of medication-related problems in 3410 participants aged 70 years and above. The review included 12 RCTs reported in 15 articles and measured the rate of hospitalisation, mortality, care home admission, medication adherence, quality of life, and medication knowledge. This systematic review found no evidence that such medication reviews reduced hospital admissions, mortality, or care home admissions. Similarly, the four studies that included quality of life (EQ-5D) found no effect of the intervention. Seven studies measured the effect on medication adherence with inconsistent results, three studies reported improvement in adherence, and four studies found no effect of the intervention.

**Almutairi et al. [17], published in 2020,** aimed to systematically review interventions that increase the appropriateness of medications used in residential aged care facilities. It included medication review, staff education, multi-disciplinary case conferencing, and computerised clinical decision support systems as interventions in 19,576 participants, with a mean age of 81 to 87 years. The review included 10 RCTs and 15 cluster-randomised controlled trials and reported medication appropriateness, hospital admissions, mortality, medication-related problems, falls, behavioural and psychological symptoms of dementia (BPSD), adverse drug events, and cognitive function as outcomes. The authors did not find any significant effect of intervention on hospital admission, mortality, fall, quality of life, cognitive function, adverse drug events, or BPSD.

**Mizokami et al. [9], published in 2019,** aimed to investigate the impact of clinical medication reviews (CMR) on reducing unplanned hospitalizations due to polypharmacy among 5299 adults between 67 to 87 years of age. The review included nine randomised controlled trials and concluded that clinical medication reviews considerably reduced the rates of unplanned hospitalizations in older patients.

**Pazan et al. [18], published in 2019,** aimed to identify the impact of pharmacological interventions on frailty or aspects of frailty in 4954 patients, aged 60 years and above. The review included 25 randomised controlled trials. A comprehensive frailty score was used in four studies, and the review concluded that no evidence for fundamental pharmacological effects on frailty could be found. Ten out of twenty-one studies showed improvement in some aspects of frailty such as physical performance and muscle strength.

**Thompson et al. [19], published in 2019,** aimed to compile the tools available to clinicians in identifying and reducing or deprescribing potentially inappropriate medications and specifically involved only frail older persons and older persons with limited life expectancy. The review included 15 tools identified from 144 articles, categorised into three main groups: model or framework for approaching deprescribing, deprescribing approach for the individual’s entire medication list, and guidance on deprescribing of individual medications. The review concluded that there are tools available to help clinicians reduce medication at various stages of the deprescribing process.

**Tjia et al. [20], published in 2013,** aimed to describe intervention studies that decreased the use of unnecessary medications in frail older adults. It included pharmacist-initiated medication reviews, academic detailing, audit and feedback reports about medication overuse, and physician-led medication reviews in 13,906 participants aged between 69 and 87 years. The review included a total of 36 studies, of which 15 were RCTs and concluded that most of the interventions that were effective in reducing inappropriate medications involved a pharmacist in the process and used an implicit criterion to identify those medications. Implicit criteria refer to a list of questions that are designed to determine medication appropriateness, as compared to explicit criteria, which refer to a list of names of medications that are targeted for discontinuation.

**Iankowitz et al. [21], published in 2012,** aimed to examine and synthesize the evidence related to the effect of computer systems' clinical decision-making tools on the frequency of ordering potentially inappropriate medications (PIMs) at discharge and related unplanned emergency room visits and hospital readmissions in community-dwelling patients older than 65 years of age. This review involved over 29,840 participants and included four RCTs and one quasi-experimental study. It concluded that clinical decision-making computer support tools, such as drug specific alerts, are effective in reducing prescribing of PIMs.

**Kaur et al. [22], published in 2009,** aimed to identify interventions that can reduce inappropriate prescribing in older adults. It included studies with over 124,802 participants between the ages of 69 to 85 years in different healthcare settings and seven types of interventions: educational, computerised support, pharmacist-based, geriatric medicine services, multidisciplinary team reviews, regulatory policies, and multi-faceted interventions. Twenty-four studies were included, of which thirteen were RCTs and two were quasi-experimental studies. The review concluded that different strategies may be useful in reducing inappropriate prescribing in older adults, although the synergistic effect of merged strategies is unclear.

**Rollason et al. [23], published in 2003,** aimed to study the effectiveness of pharmacist-led interventions in decreasing polypharmacy. It included medication reviews by pharmacists or by a team of nurses, pharmacists, and physicians in 4393 participants aged 64 to 86 years divided over eight RCTs and six controlled trials. Included studies reported a reduction in the number of medications, and the review concluded that any type of the studied intervention including a pharmacist can reduce the number of drugs that are prescribed to older patients.

### 3.5. Current Evidence of Effectiveness

The evidence outlined below serves as a guide for where the current research stands regarding the effectiveness of medication interventions against polypharmacy among frail older adults.

#### 3.5.1. Frailty Score

Only the systematic review by Pazan et al. [18] included frailty as an outcome, and only two RCTs out of twenty-five studies in that review reported substantial improvement in frailty status. The first was an RCT of a 12-week intervention of exercise training, intake of high-protein nutritional supplements, memory training, and medication review for frail older adults in the community. The second RCT enrolled patients who visited the emergency department for injuries resulting from a fall in a 3-month physical therapy program. The trial also included a 6-month screening and a follow-up programme for eye check-ups, polypharmacy, and environmental hazards. Both studies used the Short Physical Performance Battery (SPPB) for the assessment of frailty. 

#### 3.5.2. Inappropriate Medication

Six out of the ten systematic reviews focussed on inappropriate medication as an outcome. In Tjia et al. [20], 22 out of 26 included studies (85%) reported statistically significant reductions in ‘explicitly defined unnecessary medications’. Furthermore, 67% of pharmacist-initiated medication reviews, 70 % of pharmacist reviews in interdisciplinary teams, and all 5 studies on physician medication reviews reported statistically significant reductions in inappropriate medication use. 

Rollason et al. [23] reported that medication reviews by pharmacists or a team of healthcare professionals comprising pharmacists resulted in a statistically significant reduction in the number of prescribed medications in seven out of fourteen studies. However, no statistically significant effect was observed in five studies.

Kaur et al. [22] reported that educational interventions had a mixed impact on the prescription of inappropriate medications, while geriatric medicine services were effective in reducing the use of unnecessary medications. The review also observed that interventions where the prescriptions were checked by pharmacists led to a reduction in unclear and inappropriate prescriptions. 

Almutairi et al. [17] reported medication appropriateness as the main outcome and noted a significant improvement in medication appropriateness in a meta-analysis (RR 0.71; 95% confidence interval (CI): 0.60, 0.84), although with high heterogeneity (I^2^ = 91%). Specific analyses were run for the following intervention types: staff education (RR 0.66, 95% CI: 0.43, 1.01), implementation of multi-disciplinary case conferencing (RR 0.97, 95% CI: 0.92, 1.03), computerised clinical decision support systems (RR 0.78, 95% CI: 0.64, 0.95), and medication review (RR 0.62 95% CI: 0.41, 0.93).

Iankowitz et al. [21] and Thompson et al. [19] discussed tools that can be useful in the process of reducing inappropriate medications. Iankowitz et al. [21] concluded that when providers have access to computer support tools that aid in clinical decision-making, a decrease in the prescription of potentially inappropriate medications prescribed for older patients is seen. Three of the five included studies were statistically significant in favour of the intervention, and the meta-analysis based on two studies favoured the use of computer-supported clinical decision-making tools (RR 0.82; 95% CI 0.76–0.88). Thompson et al. [19] identified 15 tools for identifying and reducing or deprescribing potentially inappropriate medications that can be used for frail older individuals and those with limited life expectancy. The review concluded that these tools can be used as frameworks for managing medications by either targeting the entire list of medications or deprescribing specific medications.

#### 3.5.3. Health Care Utilisation/Hospitalisation

Four systematic reviews included hospitalisation or emergency department visits as outcomes. All reviews performed meta-analyses of the included articles.

Tasai et al. [15] reported that medication reviews performed by community pharmacists significantly reduced the risk of emergency department visits by 32% compared to usual care (RR 0.68; 95% CI 0.48–0.96), with high heterogeneity (I^2^ = 76.3%, *p* = 0.040). They also observed that the use of community pharmacists decreased the risk of hospitalizations compared with usual care (risk ratio (RR) = 0.88; 95% CI = 0.78–1.00), with no heterogeneity (I^2^ = 0.0%, *p* = 0.828). On the other hand, Abbott et al. [16] reported a rate of hospitalisation based on eight trials comprising of 2314 participants evaluating the effect of home visits performed by pharmacist for patients who were at risk of medication-related problems. No evidence of a reduction in hospital admissions was found ((RR) of 1.01 (95% CI 0.86 to 1.20, I^2^ = 69.0%, *p* = 0.89)). Almutairi et al. [17] reported hospital admissions based on eight studies comprising 10,610 residents, concluding that the interventions (e.g., pharmacist, screening tools, or education) had no effect on hospital admission (RR 1.00; 95% CI: 0.93, 1.06) with a low heterogeneity (I^2^ = 0%). Mizokami et al. [9] reported on the effect of Clinical Medication Reviews (CMRs) in older patients based on nine RCTs. Five of the studies were prescription-only reviews (Type I) or adherence reviews (Type II), and four studies were complete wide-ranging evaluations (Type III). In terms of the prevention of unplanned hospital admissions, the analysis was conducted according to a clinical setting. For inpatients, there were a total of 1487 participants in three trials, and CMR was found to significantly prevent unplanned hospitalizations (RR 0.89, 95% CI 0.80–0.98). For outpatients, a total of 3733 participants over six trials were included, but no effect was noted (RR 1.11, 95% CI 0.99–1.24). The results based on CMR types I/II and type III indicated an increase in the number of unplanned hospitalizations for types I/II (RR 1.22, 95% CI 1.07–1.38), based on 1768 participants over five trials. For CMR type III, based on 3452 participants in four trials, a significant decrease in the number of unplanned hospitalizations was observed (RR 0.86, 95% CI 0.79–0.95).

### 3.6. Reporting Quality Assessment

Table 1 below presents the seven critical domains in the AMSTAR 2 quality assessment checklist of the systematic reviews included in the current overview. 

Six reviews were of moderate quality, and four are classified as of critically low quality. All systematic reviews were considered to have conducted an adequate search, and over time, more reviews considered the risk of bias. However, only two reviews registered a protocol ahead of conducting the review, and only two reviews provided a justification for excluding studies. Appraisal of whether analytical methods were conducted appropriately suggest that, where meta-analysis is performed (n = 5), it was appropriate.

## 4. Discussion

This overview of systematic reviews summarizes the evidence on the impact of medication interventions on the management of polypharmacy in frail older adults, alongside identifying knowledge gaps. The overview collected information from ten systematic reviews published between 2003 and 2020. It included 154 studies, which comprised 100 RCTs, 15 cluster RCTs, 13 case series, 6 controlled trials, 6 pre-post studies, 4 non-randomised trials, 4 cohort studies, 3 quasi-experimental studies, 2 surveys, and 1 prospective case series. Only one systematic review, of moderate quality, evaluated a comprehensive frailty score, but with inconclusive results. Six systematic reviews reported a reduction in the prescription of potentially inappropriate medications, of which two were scored as moderate and four as critically low quality. Four systematic reviews evaluated the rate of hospital admissions as an outcome, and all were graded as moderate in quality assessment. Five systematic reviews performed a meta-analysis, while the remaining reported heterogeneity in settings and outcomes or low methodological quality of primary studies as a rationale for not performing a meta-analysis.

### 4.1. Intervention

Medication reviews performed by pharmacists alone or in a team dominated by health professionals were generally the most common type of intervention. Most systematic reviews in this overview show a positive effect of this intervention. The pharmacists are either independent prescribers and can change the prescription or identify issues with medications and recommend changes to the physician, who in turn makes the final assessment. Pharmacist-led medication interventions seemed to reduce the number of inappropriate prescriptions in a variety of settings, be it home visits, hospitals, or primary care, and led to better health outcomes in terms of reduced hospitalisation and improved frailty score. Rollason et al. [23] concluded that any type of intervention by a pharmacist can reduce the number of drugs that are prescribed to older patients. Almutairi et al. [17] also concluded that multifaceted interventions, including medication review, could improve the appropriateness of medications at care facilities. Low healthcare utilisation was also noted as a benefit resulting from medication reviews. Tasai et al. [15] and Mizokami et al. [9] concluded that clinical medication reviews for older people with polypharmacy reduced the risk of emergency department visits and unplanned hospitalisations.

Tools also play a significant role in the process of reducing inappropriate medications. Iankowitz et al. [21] concluded that a reduction in the prescription of potentially inappropriate medications occurs when clinical decision-making computer support tools, such as drug-specific alerts, are available to providers. Thompson et al. concluded that there are tools available that can help clinicians reduce medication at various stages of the deprescribing process.

### 4.2. Outcomes of Interest

**Frailty:** Pazan et al. [18] did not find consistent evidence regarding medication reviews improving frailty. Four out of the twenty-five studies included in this review studied a comprehensive frailty score. Two of those reported progress in terms of frailty [24,25], while two reported no improvement. This systematic review conducted in 2020 was the first of its kind, focussing on the impact of drug interventions on frailty or aspects of frailty. It should be noted that frailty still does not have an internationally recognised standard definition [26]. Furthermore, disagreement remains on which tools can be used to measure frailty and its severity. The commonly used frailty measurement tool ‘Clinical Frailty Scale (CFS)’ has also been recently updated [27]. Though the evidence from this review is of moderate quality, it provides inconclusive evidence of medication review on frailty, amplifying the need for more comprehensive RCTs on this subject.

**Inappropriate medication:** Tjia et al. [20], Rollason et al. [23], and Kaur et al. [22] reported significant reductions in the number of prescribed medications. Among these, only Tjia et al.’s [20] review, conducted in 2013, is of moderate quality, while the other two reviews are of critically low quality, thus dampening the overall quality of evidence regarding the effect of medication reviews on the reduction in inappropriate medications. Rollason et al. [23] and Tjia et al. [20] also concluded that more research is needed on reducing unnecessary medications in frail older adults or those approaching end of life. Kaur et al. showed that the evidence was unclear about the collaborative effect of combined approaches undertaken at the same time, thus highlighting the need to prioritise older people who are living with frailty and polypharmacy for medication reviews and deprescribing interventions to improve their health-related outcomes. 

**Hospitalisation:** Abbot et al. [16] and Almutairi et al. [17], based on eight trials each, found no evidence that medication reviews reduced hospital admissions or mortality. However, Tasai et al. [15] reported that medication reviews significantly reduced the risk of emergency department visits and decreased the risk of hospitalizations. Mizokami et al. [9], on the other hand, reported results regarding the type of clinical medication reviews in inpatient and outpatient settings based on nine trials. A reduction in hospitalisations was noted for Type III CMR (wide-ranging evaluation), as compared to Types I and II. In addition, interventions were found to be effective in inpatient settings but not in outpatient settings. All the systematic reviews were of moderate quality and published within the last two years. Though moderate quality evidence can be used to support clinical decision making, as the results are inconsistent, further research is needed to specifically study the impact of medication reviews on hospitalisation among frail older adults.

### 4.3. Quality of the Evidence

The confidence in the results generated by an overview largely depends on the quality of the included systematic reviews. The quality of the systematic reviews included in this overview varies, with six systematic reviews rated as moderate and four rated as critically low, using the AMSTAR 2 checklist. None of the included systematic reviews achieved a high-quality rating. This illustrates an inconsistency in the quality of the evidence regarding medication interventions, and while no reviews were excluded based on their quality, the methodological limitations prevent us from drawing definitive conclusions and imply that the evidence derived from this overview must be interpreted with caution. There is a need for reviews with higher methodological quality in this area.

### 4.4. Strengths and Limitations

The objective of this overview was to assess the impact of medication interventions that target polypharmacy in frail older adults, which hopefully would encourage a balanced use of medications among frail older adults. The strength of this overview is primarily the focus on interventions that have used clinically relevant outcomes, such as the reduction in the prescription of potentially inappropriate medications and hospitalisation. The assessment of the methodological quality of the systematic reviews and the fact that no restriction was applied regarding the date of publication are additional strengths.

Overviews of systematic reviews are likely to be limited, especially when attempting multifaceted subjects and when there are heterogeneous results [28]. In organising the results of 154 primary studies, characteristics of individual studies may have been overlooked. One of the most important limitations of this overview is in the methodology section, as the second stage of screening and study selection process, data extraction, and quality assessment were performed by only one person/researcher, leading to a possible risk of bias [29].

The search strategy was initially devised to capture a wider range of results, keeping the British Geriatric Society (BGS) Frailty syndromes in consideration, as this overview is a part of a larger project being conducted on frailty by Trinity College Dublin for the Public Health Agency, Ireland. The objective of the search was to build a comprehensive database on frailty or one of the frailty syndromes described by BGS. Thus, it is possible that a different search strategy specifically developed for this overview could have identified a different set of reviews. Furthermore, the comprehensive search was conducted only in EMBASE. Although EMBASE includes all records from MEDLINE, there is a risk that we have failed to identify all relevant reviews. In addition, many studies are included multiple times in different systematic reviews, leading to repetition and research waste.

### 4.5. Implications for Practice and Recommendations for Future Research

Medication reviews, by pharmacists alone or by a team of health professionals, have presented favourable results. Nonetheless, the acceptance of these reviews and suggestions by the practising clinicians play a significant role in the success of such reviews. It is apparent that medication interventions by pharmacists are likely to improve the prescription of medicines in frail older adults. This review also emphasises the significance of developing a clinical role that pharmacists can play when integrated with other health practitioners. Alongside medication reviews, the concept of medication management has also been suggested, which could enable a better understanding of frailty and polypharmacy [30]. Studies have shown that medication management reduces hospital readmission for older people [31].

To reinforce the evidence and support the implementation of medication interventions, it is important to make available a detailed description of the settings in which the interventions were conducted and the results that were achieved, so that they can be reproduced in diverse contexts. Furthermore, the cost of these medication/pharmacological interventions must be compared with the economic burden of inappropriate medications. Qualitative studies that involve both patients and healthcare practitioners could help in understanding the barriers regarding the implementation of medication interventions.

## 5. Conclusions

The systematic reviews included in this overview clearly show the advantage of pharmacist-led medication reviews in reducing the number of inappropriate medications administered to frail older adults, but the quality of the evidence was moderate to critically low. The evidence on frailty was inconclusive, with only one systematic review presenting it as an outcome. Likewise, the evidence on hospitalisations was mixed, with only one systematic review finding a reduction in hospitalisation and emergency department visits. Further research investigating the causal relationship between polypharmacy and frailty syndromes is required to identify ways to reduce hospitalisations in frail older adults.

## Figures and Tables

**Table 1:** AMSTAR; illustration of 7 critical domains (in reverse chronological order).

Author, Year	Protocol Registered (Item 2)	Adequacy Search (Item 4)	Justification for Excluding Studies (Item 7)	Risk of Bias Assessed (Item 9)	Appropriateness of Analytic Methods (Item 11)	Risk of Bias Considered in Interpreting Results (Item 13)	Assessment of Publication Bias (Item 15)	Overall AMSTAR Rating
Abbott et al., 2020 [16]	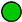	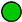	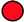	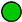	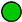	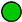	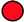	Moderate
Almutairi et al., 2020 [17]	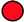	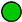	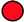	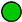	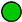	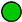	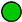	Moderate
Pazan et al., 2020 [18]	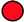	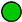	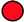	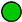	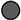	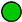	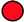	Moderate
Mizokami et al., 2019 [9]	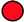	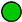	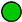	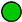	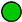	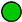	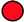	Moderate
Tasai et al., 2019 [15]	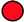	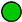	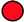	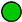	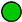	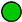	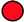	Moderate
Thompson et al., 2019 [19]	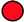	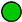	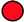	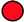	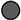	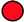	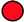	Critically Low
Tjia et al., 2013 [20]	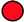	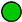	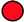	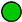	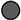	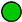	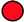	Moderate
Iankowitz et al., 2012 [21]	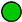	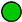	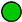	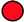	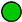	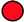	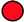	Critically Low
Kaur et al., 2009 [22]	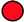	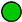	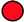	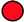	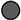	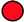	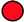	Critically Low
Rollason et al., 2003 [23]	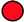	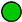	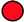	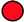	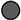	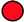	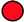	Critically Low

Note: Green dot means Yes, red dot means No, and black dot means no meta-analysis was conducted.

## Data Availability

Not applicable.

## Supplementary Materials

The following supporting information can be downloaded at https://www.mdpi.com/article/10.3390/jcm12041379/s1: Table S1: Results of Stage 1 indicating types of included studies by specified dimension of health category and type of research study. As systematic reviews may consider more than one health category or study type, reviews may appear more than once.
