# Supplementary material for: An Overview of Systematic Reviews and Meta-Analyses on the Effect of Medication Interventions Targeting Polypharmacy for Frail Older Adults
Source: J Clin Med. 2023 Feb 9;12(4):1379. doi: 10.3390/jcm12041379 (PMC9960328; doi:10.3390/jcm12041379)
Supplement: Supplementary file 1 [file jcm-12-01379-s001.zip › jcm-2079346-supplementary.pdf]

Supplementary materials

Search terms

Frailty (and Medication) Systematic Reviews in EMBASE - 2711 Results

'meta analysis'/exp
((meta NEXT/1 analy*) or metaanalys*):ti,ab
(systematic NEXT/1 (review* or overview*)):ti,ab
#1 OR #2 OR #3
cancerlit:ab
Cochrane:ab
embase:ab
(psychlit or psyclit):ab
(psychinfo or psycinfo):ab
(cinahl or cinhal):ab
'science citation index':ab
bids:ab
#5 OR #6 OR #7 OR #8 OR #9 OR #10 OR #11 OR #12
'reference lists':ab
bibliograph*:ab
'hand-search*':ab
'manual search*':ab
'relevant journals':ab
#14 OR #15 OR #16 OR #18
'data extraction':ab
'selection criteria':ab
#20 or #21
review:it
#22 and #23
letter:it
editorial:it
'animal'/de
'human'/de
#27 not (#27 and #28)
#25 OR #26 OR #29
#4 or #13 or #19 or #24
#31 not #30
'frail elderly'/exp OR 'frailty'/exp
(frail* or pre-frail* or elder*):ab,ti
(deficit* NEAR/3 (accumulat* OR cumulative)):ti,ab
#33 or #34 OR #35
'drug therapy'/exp OR 'polypharmacy'/exp OR 'prescription'/exp OR 'prescription drug'/exp
('drug therap*' OR 'drug treatment?' OR 'medicament therap*' OR 'medicament treatment?' OR 'medication?' OR 'medicinal therapy' OR 'medicinal treatment' OR 'pharmaceutical therap*' OR 'pharmaceutical treatment?' OR 'pharmaco-therap*' OR 'pharmaco-treatment?' OR 'pharmacological therap*' OR 'pharmacological treatment?' OR 'pharmacotherap*' OR 'pharmacotreatment?'):ti,ab
('multi-drug therap*' OR 'multidrug therap*' OR 'multiple drug therap*' OR 'multiple drug treatment?' OR 'multiple pharmacotherapy' OR 'poly pharmacy' OR 'polypharmacy' OR 'polypragmasia' OR 'polypragmasy'):ti,ab
('drug prescribing' OR 'drug prescription' OR 'drug prescriptions' OR 'prescription' OR 'prescriptions'):ti,ab
'prescription drug':ti,ab
#37 OR #38 OR #39 OR #40 OR #41
#32 AND #36 AND #42

Table S1: Results of Stage 1 indicating types of included studies by specified dimension of health category and type of research study. As systematic reviews may consider more than one health category or study type, reviews may appear more than once.

**Types of research studies** / **Health category**	Prevalence/ Incidence	Identification/ Diagnostic tests	Experimental (RCTs/Quasi-RCTs)	Epidemiology (risk to develop)	Epidemiology (sequalae/implications)	Cost of illness	Economic Evaluation	Other study types
Frailty	82	111	141	117	131	4	8	29
Delirium	2	7	26	14	16	0	1	2
Falls	5	8	68	32	41	1	7	4
Immobility	1	0	3	4	4	0	0	0
Incontinence	3	0	8	1	5	0	0	2
Susceptibility to side effects of medication	8	4	**24**	15	22	0	2	**4**
Loneliness and isolation	1	1	1	0	8	0	0	6
Nutrition	8	4	41	21	16	0	0	2

Bold indicates reviews selected for the current overview
